# Epidemiological characterization and prognostic factors in patients with confirmed cerebral cryptococcosis in central Taiwan

**DOI:** 10.1186/s40409-015-0012-0

**Published:** 2015-05-12

**Authors:** Chang-Hua Chen, Hiu-Ngar Sy, Li-Jhen Lin, Hua-Cheg Yen, Shao-Hung Wang, Wei-Liang Chen, Yu-Min Chen, Yu-Jun Chang

**Affiliations:** Division of Infectious Disease, Department of Internal Medicine, Changhua Christian Hospital, Changhua, 135 Nanhsiao Street, Changhua, Taiwan; Department of Neurology, Changhua Christian Hospital, Changhua, Taiwan; Infection Control Committee, Changhua Christian Hospital, Changhua, Taiwan; Department of Neurosurgery, Changhua Christian Hospital, Changhua, Taiwan; Department of Microbiology, Immunology and Biopharmaceuticals, National Chiayi University, Chiayi City, Taiwan; Department of Medical Imaging, Changhua Christian Hospital, Changhua, Taiwan; Department of Pharmacy, Changhua Christian Hospital, Changhua, Taiwan; Laboratory of Epidemiology and Biostatistics, Changhua Christian Hospital, Changhua, Taiwan; Department of Nursing, College of Medicine & Nursing, Hung Kuang University, Taichung, Taiwan

**Keywords:** Cryptococcus neoformans, Cryptococcus gattii Cryptococcosis, Meningitis, Outcome, Risk Assessment

## Abstract

**Background:**

Cryptococcal meningitis is a deadly fungal infection. This study aimed to characterize the epidemiology of cerebral cryptococcosis and to define its prognostic factors.

**Methods:**

This cross-sectional study collected clinical information from cryptococcal meningitis patients with confirmed cerebral cryptococcosis from 2006 to 2012 at the Changhua Christian Healthcare System to access prognostic factors.

**Results:**

Fifty-nine adult cryptococcal meningitis patients were studied. The incidence at Changhua Christian Healthcare System was approximately 170 episodes per 100,000 patients within the studied period. Forty-one of 59 cryptococcal meningitis patients developed complications. Overall, 12 of 59 patients died, for a three-month mortality rate of 20.3 %. Prognostic factors positively associated with the three-month mortality included age (>55 years), patient delay, prolonged delay by the doctor in administering antifungal agent therapy, duration of intensive care unit stay, chronic lung disease, cryptococcemia, headache, altered mental status, positive blood cultures, and high cerebrospinal fluid opening pressure (≥250 mm H_2_O).

**Conclusions:**

We strongly recommend early administration of an antifungal agent to each suspected cryptococcal meningitis patient to decrease both the delay by doctors in administering therapy and the mortality risk. Aggressive and supportive care for severe cryptococcal meningitis patients is critical to decrease overall mortality from this infection.

## Background

*Cryptococcus neoformans-Cryptococcus gattii* species complex is the ubiquitous encapsulated yeast that causes significant infections, ranging from asymptomatic pulmonary colonization to life-threatening meningoencephalitis [1]. Cryptococcosis is a severe opportunistic fungal infection causing morbidity and mortality and can manifest as meningitis, pneumonia, or cryptococcemia in both immunocompetent and immunocompromised hosts [2–5]. It is estimated that approximately one million cases and 625,000 deaths occur annually worldwide from cryptococcal meningitis (CM) among HIV-infected individuals, but only a few studies have estimated the incidence of CM among non-HIV-infected patients [3, 6]. CM is not uncommon in Taiwan, and 845 new cases were detected using the National Health Insurance Research Database between January 2000 and December 2007 [7]. Tseng *et al*. [4] reported that patients infected with *C. gattii* were more likely to reside in central Taiwan than those infected with *C. neoformans*, but there are few geographic evaluations of CM in central Taiwan.

Outcomes and treatment failure are usually associated with underlying conditions, a delay in diagnosis or absence of treatment with a fungicidal drug [8, 9]. However, prognostic factors have mostly resulted from reports of clinical trials conducted in earlier treatment periods and risks [3, 10–13]. Because medical and surgical advancements may not serve the rapidly growing high-risk population of patients, more data on the current epidemiology and prognostic factors of CM are necessary.

Although many studies have evaluated CM, some lack confirmed case studies, contain only mycological data, only focus on HIV or post-transplantation status, or do not provide incidence estimates [2, 3, 5, 6, 9, 14, 15]. There has been no comprehensive study providing a risk assessment for CM. Furthermore, the underlying mechanisms of CM are not yet fully understood, and so the most effective means of prevention remains unknown.

CM infection is fatal without treatment. As a result, rapid recognition, diagnosis, and treatment are required. Little is known about CM in the central Taiwan population. Therefore, this study reports a retrospective cross-sectional study for patients with confirmed CM at Changhua Christian Healthcare System (CCHS) between 2006 and 2012 with two specific aims: to characterize the epidemiology of cerebral cryptococcosis at CCHS and to define prognostic factors associated with death from CM.

## Methods

### Setting and population

The population of Changhua, Nantou, and Yunlin counties is mostly served by the CCHS, which has maintained comprehensive clinical records since 1867. These three counties are located in central Taiwan and had a population of 1,362,563 in 2014. CCHS is a 3200-bed medical institute in central Taiwan. Changhua Christian Hospital, part of the CCHS, is a 1800-bed tertiary referral medical center located in northern Changhua County and has been a Joint Commission International–accredited hospital since 2009.

### Microbiological evaluation, serological examinations, and antifungal susceptibility testing of *Cryptococcus* isolates

Cerebrospinal fluid (CSF) samples were routinely sent for tests to obtain complete white blood cell counts and differential counts, glucose and protein concentrations, and India ink stain and cultures. Urine, pus, lung, ascites, pleural effusion, lymph node, and/or other biopsy tissues and aspiration specimens were collected and submitted for culturing when indicated. Serum and CSF cryptococcal antigen titers were determined using commercial latex agglutination tests (*Cryptococcus* Antigen Latex Agglutination Test System, Immuno-Mycologics, Inc., USA). There was no difference in the culture and identification systems during the study period.

The blood and CSF specimens were cultured in the BD BACTEC FX (Becton Dickinson, USA) culture system, which is fully automated for microorganism growth detection. A yeast-like microorganism was detected by microscopic inspection after gram staining, and the growth characteristics were observed on agar plates. Further identification was performed using the API-32C (BioMerieux, France) yeast identification system. All blood isolates were stored at −80 °C in a Thermo Scientific Forma (Thermo Fisher Scientific, USA) 900 series 955 model.

For drug susceptibility testing, all available stored isolates were thawed, inoculated in Sabouraud dextrose agar, and incubated at 35 °C for 72 h. The procedures were done according to the manufacturer’s Sensititre YeastOne protocol (TREK Diagnostic Systems, UK) [16]. Briefly, yeast colonies from the Sabouraud dextrose agar plates were placed into test tubes containing 10 μL normal saline and vortexed; the turbidity of the suspensions was adjusted to a 0.5 McFarland standard. The suspensions were then placed into the panel wells and incubated in a 35 °C incubator for 72 h. A control well without an antifungal agent (AFA) was used to ensure the quality of growth and purity. After incubation, the control suspension was subcultured onto another Sabouraud dextrose agar plate to ensure the growth of 10-80 pure colonies for each corresponding candida isolate. The panel wells were read and interpreted automatically using a Vizion instrument (Thermo Scientific Sensititre Vizion Digital MIC Viewing System, Thermo Fisher Scientific, UK).

### Definitions

CM was defined as a CSF culture positive for *C. neoformans-C. gattii* species complex in this study. Time until diagnosis was defined by the duration of symptoms (in days), that is, the number of days between symptom onset and meeting of the case definition. Patient delay was defined as the interval between the onset of symptoms and initial presentation to the hospital. Delay by the doctor in administering AFA therapy was defined as the interval between the initial presentation to the hospital and the start of AFA. Prolonged delay was defined as a delay greater than the median. Delay by the doctor in administering steroid therapy was defined as the interval between the initial presentation to the hospital and the start of steroid therapy. Prolonged delay was defined as a delay greater than the median. The patients were divided into two groups: the deceased group (comprising patients who died within three months of CM diagnosis) and the survival group (comprising those still living after three months). The median of prolonged delay of AFA was six days. In terms of AFA administration, patients were divided into two groups: the delayed group was defined as those patients who received AFA more than six days after admission and the non-delayed group was defined as those patients who received AFA within six days of admission.

### Case findings and analysis

Diagnosis of cryptococcosis was defined in this study according to a review of microbiological reports, *The International Classification of Diseases*, 9^th^ revision (ICD-9). Cases were sorted in computerized indices by ICD-9 diagnosis in codes 117.5 and 321.0. The medical charts of CCHS employed were from January 1, 2006, to December 31, 2012 [17]. CM was defined as a CSF culture positive for *C. neoformans-C. gattii* species complex in this study, and the definitive diagnosis of CM was established based on the presence of symptoms and/or signs suggestive of meningitis, a CSF culture positive for *C. neoformans-C.* gattii species complex, and a CSF smear positive for India ink. Brain computed tomography and/or magnetic resonance imaging studies were performed on admission, at the discretion of the physician; and follow-up brain images were dependent on the physician’s decision.

Between January 2006 and December 2012, hospitalized patients aged more than 18 years and diagnosed with CM were recruited. Each patient had a medical chart, which contained medical diagnoses, surgical interventions, and other key information from the medical records. The medical records of all CM cases were manually reviewed by the primary investigator (CCH) to confirm the diagnosis using CCHS resources. Cases that were judged problematic were reviewed by the secondary investigator (SSY). Only the first CM episode in each person during the study period was included for statistical analyses. To calculate disease incidence, the entire admitted patient population of CCHS was considered at risk for infection. All *Cryptococcus* isolates cultured from CSF were considered clinically significant. CM was defined as the isolation of *Cryptococcus* from CSF in an adult patient (age ≥ 18 years) between January 1, 2006, and December 31, 2012. We excluded those patients who presented the following conditions: less than 18 years old, polymicrobial bacteremia (*C. neoformans-C. gattii* species complex with another pathogen), incomplete data, or inconsistency between the data from, ICD-9 diagnosis codes 117.5 and 321.0 and the microbiological dataset.

### Validity and reliability of methods

A small-scale validity study was performed on the case-finding procedures. The validity of the system was tested by a review of the complete medical charts of 59 individuals who did not have any relevant code in ICD-9; records were obtained from the microbiological dataset on or before December 31, 2012 (the end of the study period). To examine the reliability of both the case-finding procedure and the diagnoses, all cases judged problematic by the infectious disease specialist (CCH) were evaluated. Among the 65 cases examined, the panel accepted 59 diagnoses (91 %) and revised six.

### Evaluation and investigation

A standardized case report form was used to collect data on age, sex, site of involvement, underlying disease, clinical presentation, length of symptoms, diagnostic laboratory and radiographic reports, medical treatment, interventions, and outcomes [17]. Severity of stroke was defined according to the Canadian Neurological Scale [18]. Concomitant diseases included hypertension, chronic kidney disease, diabetes, hyperlipidemia, preexisting cardiovascular disease, and previous stroke. Hypertension, chronic kidney disease, diabetes, and hyperlipidemia were defined according to previous recommendations [19–22]. Stroke, heart failure, atrial fibrillation, and congestive heart failure were identified by taking a history and by reviewing the medical chart.

### Treatment and follow-up

AFA was classified according to drugs administered for induction, consolidation, and chronic suppression [17]. Induction was defined by the initial drug given for more than three days [17]. Consolidation was defined as the therapy given following a response to the induction regimen [17]. Chronic suppression represented the ongoing maintenance of AFA given to patients without evidence of active disease [17]. The duration of AFA and the daily dose were recorded; and the primary outcome was the three-month mortality in this study. Mortality attributable to cryptococcosis was also analyzed. A follow-up record was maintained from the date of diagnosis to the date of death or to the date of the last available record. For the statistical analysis, mild to moderate sequelae and severe sequelae were regarded as poor brain outcomes.

### Statistical analysis

Clinical, neuroradiological, and CSF data were compared between groups. The *χ*^2^ or Fisher’s exact test was used for binary variables, and Student *t*-test was employed for age comparisons between the two groups. Because patient delay, delay by the doctor in administering AFA, delay by the doctor in administering corticosteroid therapy, and total delay were not normally distributed, the nonparametric Mann–Whitney test was used to compare delay differences between groups. Multivariable analyses for prognostic factors related to three-month mortality were performed using multiple logistic regression analysis. Odds ratios (ORs) and their corresponding 95 % confidence intervals (95 % CIs) were also estimated using ordinary logistic regression. After univariate estimates were calculated, ORs were obtained in multivariate models for all independent variables. A p value < 0.05 was considered statistically significant. All data were analyzed using the software SPSS (version 17).

## Results and discussion

### Patient characteristics: laboratory and radiographic findings

Sixty-five CM patients were enrolled during the study period, but because of incomplete data and inconsistent diagnosis, only 59 were analyzed. The incidence of CM in adults consulting CCHS was not increased, ranging from 168.9/100,000 patient in 2006 to 172.3/100,000 patient in 2012. There was no significant linear trend for an annual increase in CM across the study period. Of the 59 patients analyzed (Table 1), 43 (72.9 %) had diabetes mellitus, 12 (20.3 %) were HIV-positive, and 32 (54.2 %) had malignancies. Cryptococcemia was noted in 21 (35.6 %) and pulmonary cryptococcosis in 21 (35.6 %) patients.

**Table 1:** Comparison of key variables between the survivor and deceased groups of CM patients

Variables	Survivor group	Deceased group	Total		Univariate logistic regression analysis		
	*n* = 47 (%)	*n* = 12 (%)	*n* = 59	p value	OR	95 % CI	p value
Age > 55 years	18	2	20	0.192	1.077	1.013–1.146	0.019
Gender – male	28 (59.6)	10 (83.6)	38	0.182			
Prior cryptococcal infection, not related to this episode	11 (23.4)	2 (16.7)	13	0.762			
Length of stay in the ICU (days), median (range)	5 (1–21)	8 (1–48)	–	<0.001	5.217	1.030-26.425	0.046
Underlying disease							
DM	34 (72.3)	9 (75.0)	43	0.052			
HTN	31 (66.0)	6 (50.0)	37	0.334			
Chronic lung disease	20 (42.6)	9 (75.0)	29	0.024	0.34	0.100-1.240	0.09
Hyperlipidemia	26 (55.3)	6 (50.0)	32	0.102			
Previous TB infection	2 (4.3)	1 (8.3)	3	0.203			
CV disease	26 (55.3)	4 (33.3)	30	1			
Renal disease	16 (34.0)	4 (33.3)	20	0.102			
Malignancy, other than hematological malignancy	26 (55.3)	6 (50.0)	32	0.102			
Hematological malignancy	25 (53.2)	5 (41.7)	30	0.181			
Hematological disease	23 (48.9)	5 (41.7)	28	1			
Autoimmune disease	23 (48.9)	3 (25.0)	26	0.591			
Psychiatric disease	16 (34.0)	5 (41.7)	21	1			
HIV	10 (21.3)	2 (16.7)	12	1			
Site of infection							
Pulmonary cryptococcosis	13 (27.7)	8 (66.7)	21	0.074	0.44	0.180–1.070	0.07
Cryptococcemia	12 (25.5)	9 (75.0)	21	0.032	5.09	2.540–10.220	<0.001
Clinical presentation							
Fever	15 (31.9)	6 (50.0)	21	0.315			
Malaise	13 (27.7)	2 (16.7)	15	0.381			
Weight loss	12 (25.5)	1 (8.3)	13	0.281			
Headache	24 (51.1)	2 (16.7)	26	0.031	3.3	0.696-15.642	0.133
Visual change	7 (14.9)	1 (8.3)	8	0.67			
Cranial nerve palsy	2 (4.3)	2 (16.7)	4	1			
Cough	10 (21.3)	2 (16.7)	12	1			
Dyspnea	9 (19.1)	1 (8.3)	10	1			
Altered mental status	12 (25.5)	8 (66.7)	20	0.005	4.09	1.540-9.220	<0.001
Abdominal pain	42 (89.4)	8 (66.7)	50	0.083			
Decreased urine output	24 (51.1)	4 (33.3)	28	0.109			
Diagnostic criteria							
Serum CrAg 1:512	13 (27.7)	2 (16.7)	15	0.381			
CSF CrAg >1:512	10 (21.3)	4 (33.3)	14	0.281			
CSF white cell count (cell/mm^3^) (mean, SD)	83.4 ± 32.6	80.4 ± 30.6	–	0.849			
CSF % lymphocytes (mean, SD)	207.4 ± 109.8	177.4 ± 82.8	–	0.781			
CSF protein (mg/dL; mean, SD)	0.25 ± 0.22	0.22 ± 0.20	–	0.35			
CSF blood glucose (mean, SD)	−94 ± 175.1	−90 ± 155.2	–	0.674			
CSF response (day 14 vs. day 1; mean, SD)	83.4 ± 32.6	80.4 ± 30.6	–	0.943			
CSF opening pressure > 250 mm H_2_O	12 (25.5)	6 (50.0)	18	0.032	2.93	1.250-6.880	0.023
Neuroradiological findings							
Basal meningeal enhancement	6 (12.8)	4 (33.3)	10	0.059			
Hydrocephalus	20 (42.6)	9 (75.0)	29	0.117			
Cryptococcoma	4 (8.5)	2 (16.7)	6	0.589			
Neurosurgical intervention	5 (10.6)	6 (50.0)	11	0.147			
Stroke	4 (8.5)	2 (16.7)	6	0.591			

CI: confidence interval; CM: cryptococcal meningitis; CrAg: cryptococcal antigen; CSF: cerebrospinal fluid; CV: cardiovascular; DM: diabetes mellitus; HIV: human immunodeficiency virus; HTN: hypertension; ICU: intensive care unit; OR: odds ratio; SD: standard deviation; TB: tuberculosis

Before admission, 26 (44.1 %) patients exhibited symptomatic headache, 21 (35.6 %) had fever, and 20 (33.9 %) had altered mental status. Twenty-six (44.1 %) patients showed a high CSF opening pressure (>180 mm H_2_O), and 18 (30.5 %) patients showed a CSF opening pressure higher than 250 mm H_2_O. Every patient received AFA. Thirty-nine (66.1 %) patients received amphotericin B deoxycholate (deoxy-AmB) or lipid formulation amphotericin B (lipid-AmB) with flucytosine (5-FC), 14 (23.7 %) patients received deoxy-AmB or lipid-AmB with fluconazole (FLU), and six (10.2 %) patients received FLU. Forty-one (69.5 %) CM patients developed complications, including 41 with respiratory failure, 29 with hydrocephalus, 19 with hepatitis, and six with stroke. Eleven (44 %) of the 25 patients with hydrocephalus underwent an operation. Overall, 12 of the 59 CM patients died, for an overall mortality rate of 20.3 %.

Of the 59 CM patients, a cryptococcal antigen (CrAg) titer from either serum or CSF was elevated in every patient (Table 1). Fifteen (25.4 %) and 14 (23.7 %) patients showed elevated titers (>1:512) in serum and CSF, respectively. Median serum CrAg was 1:256 (interquartile range 1:16–1:2,048) and median CSF CrAg was 1:256 (interquartile range 1:16–1:2,048). The proportion of patients with positive CSF cultures was similar across groups, 82-86 % (p = 0.722). All 59 CM patients received a head computed tomography or brain magnetic resonance imaging; 29 (49.2 %) patients demonstrated hydrocephalus, 6 (10.2 %) had cryptococcoma, 10 (17.0 %) had basal meningeal enhancement, and 14 (23.7 %) were normal.

As we know, this is the first study to evaluate the risk factors and clinical outcomes of CM patients in central Taiwan.

### Treatment and outcomes

As to induction therapy, 39 (66.1 %) patients received a combination of either deoxy-AmB or lipid-AmB plus 5-FC. Fourteen (23.7 %) patients received a combination of deoxy-AmB or lipid-AmB plus FLU. Finally, six (10.2 %) received FLU alone. Most patients received a 200–400 mg average daily dose of FLU. Comparison of induction regimens with combination therapy, as measured by the three-month mortality, is presented in Table 2.

**Table 2:** Comparison of induction regimens with combination therapy between survival and deceased groups of CM patients

Variables	Survivor group	Deceased group	Total		Univariate logistic regression analysis		
	*n* = 47 (%)	*n* = 12 (%)	*n* = 59	p value	OR	95 % CI	p value
Mean age (years ± SD)	53.5 ± 17.8	69.2 ± 15.6	56.7 ± 18.4				
Age > 55 years	32 (68.1)	10 (83.3)	43	0.019	1.07	1.013-1.146	0.019
Gender – male	18 (38.3)	2 (16.7)	20	0.182			
Patient delay	28 (59.6)	10 (83.3)	38	0.012	1.87	1.274-11.230	0.015
Patient delay (days), median (range)	10 (1–124)	9 (1–91)	10 (1–124)	0.389			
Delay by the doctor in administering AFA	32 (68.1)	4 (33.3)	36	0.235			
Delay by the doctor in administering AFA (days), median (range)	6 (1–180)	4 (1–28)	6 (1–180)	0.485			
Prolonged delay by the doctor in administering AFA therapy (>6 days)	16 (34.0)	7 (58.3)	23	0.009	1.45	1.080-12.070	0.008
Delay by the doctor in administering corticosteroid	18 (38.3)	5 (41.7)	23	0.062			
Delay by the doctor in administering corticosteroid (days), median (range)	8 (1–14)	9 (1–28)	8 (1–28)	0.389			
Total delay	32 (68.1)	10 (83.3)	42	0.055			
Total delay (days), median (range)	19 (1–180)	17 (1–91)	19 (1–180)	0.459			
Prior cryptococcal infection, not related to this episode	11 (23.4)	2 (16.7)	13	0.762			
Length of stay in the ICU (days), median (range)	5 (1–21)	8 (1–28)	5 (1–28)	<0.001	4.217	1.030-23.025	0.034
Diagnostic criteria							
Serum CrAg >1:512	13 (27.7)	2 (16.7)	15	0.381			
CSF CrAg >1:512	10 (21.3)	4 (33.3)	14	0.281			
Initial CSF WBC count per mm^3^, median (range)	9 (0–51)	20 (0–250)	106	0.056			
CSF opening pressure > 250 mm H_2_O	12 (25.5)	6 (50.0)	18	0.032	2.93	1.250-6.880	0.013
Minimum inhibitory concentration of amphotericin B > 0.5 μg/mL	1 (2.1)	0	1	0.559			
Minimum inhibitory concentration of FLU >8 μg/mL	1 (2.1)	0	1	0.862			
On AFA							
Deoxy-AmB or lipid-AmB + 5-FC	33 (70.2)	6 (50.0)	39	0.005	1		
Deoxy-AmB or lipid-AmB + FLU	10 (21.3)	4 (33.3)	14	–	3.3	1.696-15.642	0.133
FLU	2 (4.3)	4 (33.3)	6	–	16.5	2.257-120.639	0.006
Complications							
Respiratory failure	29 (61.7)	12 (100.0)	41	<0.001	5.217	1.030-26.425	0.046
Hydrocephalus	20 (42.6)	9 (75.0)	29	0.117			
Neurosurgical intervention	5 (10.6)	6 (50.0)	11	0.147			
Hepatitis	15 (31.9)	4 (33.3)	19	1			
Stroke	4 (8.5)	2	6	0.591			

5-FC: flucytosine; AFA: antifungal agent; deoxy-AmB: amphotericin B deoxycholate; CI: confidence interval; CM: cryptococcal meningitis; CrAg: cryptococcal antigen; CSF: cerebrospinal fluid; FLU: fluconazole; ICU: intensive care unit; lipid-AmB: lipid formulation amphotericin B; OR: odds ratio; SD: standard deviation

In this study, 20.3 % of CM patients died within three months, which is a lower percentage than that in Tseng’s previous report [5]. The present study is a large, single-site, cross-sectional series of well-described, confirmed CM cases in a population that focuses on the prognostic factors associated with mortality. Factors observed associated with mortality included monotherapy, respiratory failure, cryptococcemia, length of stay in the ICU, baseline high CSF opening pressure (≥250 mm H_2_O), patient delay, prolonged delay by the doctor in administering AFA, and age (>55 years). On the other hand, mortality was not related to neuroradiological findings (basal meningeal enhancement, hydrocephalus, and cryptococcoma) or CSF CrAg titer, cerebral stoke, or antifungal susceptibility tests.

### Prognostic factors

Table 1 summarizes the important clinical, neuroradiological, and CSF findings in both groups. A three-month mortality rate was 20.3 % among all patients. Twenty deaths (60 %) were attributable to cryptococcosis, according to available data. In univariate analyses, prognostic factors associated with three-month mortality included age (>55 years) (*p* = 0.192), length of stay in the intensive care unit (ICU; *p* < 0.001), chronic lung disease (*p* = 0.024), cryptococcemia (*p* = 0.032), headache (*p* = 0.031), altered mental status (*p* = 0.005), and high CSF opening pressure (≥250 mm H_2_O) (*p* = 0.032). In multivariate logistic regression analyses, monotherapy (OR 16.5, 95 % CI 2.257–120.639, *p* = 0.006), respiratory failure (OR 5.217, 95 % CI 1.030–26.425, *p* = 0.046), cryptococcemia (OR 5.09, 95 % CI 2.540–10.220, *p* < 0.001), ICU length of stay (OR 4.217, 95 % CI 1.030–23.025, *p* = 0.034), baseline high CSF opening pressure (≥250 mm H_2_O) (OR 2.93, 95 % CI 1.25–6.88, *p* = 0.013), and age (>55 years) (OR 1.07, 95 % CI 1.030–1.146, *p* = 0.019) were associated with increased odds of mortality.

Table 2 summarizes the risk factors for mortality in both groups. The median delay by the doctor in administering AFA therapy for all patients was six days, and the median delay by the doctor in administering corticosteroid therapy among corticosteroid-treated patients was eight days; these values were set as the cutoff points to define prolonged delays by the doctors.

There was no difference in the durations of delay by the doctors in administering AFA therapy (*p* = 0.484) or corticosteroids (*p* = 0.022) between the two groups. After univariate logistic regression analysis, the OR for monotherapy (OR 16.5, 95 % CI 2.257–120.639, *p* = 0.006), respiratory failure (OR 5.217, 95 % CI 1.030–26.425, *p* = 0.046), cryptococcemia (OR 5.09, 95 % CI 2.540–10.220, *p* < 0.001), length of stay in the ICU (OR 4.217, 95 % CI 1.030–23.025, *p* = 0.034), baseline high CSF opening pressure (≥250 mm H_2_O) (OR 2.93, 95 % CI 1.250–6.880, *p* = 0.013), patient delay (OR 1.87, 95 % CI 1.274–11.230, *p* = 0.008), prolonged delay by the doctor in administering AFA (OR 1.45, 95 % CI 1.080–12.070, *p* = 0.008), and age (>55 years) (OR 1.07, 95 % CI 1.030–1.146, *p* = 0.019) were significantly higher in the deceased group.

Patients older than 55 years and those presenting with headache, altered mental status, and high CSF opening pressure had an increased risk of death. The association between high intracranial pressure and mortality was expected because aggressive management of high intracranial pressure is known to be crucial. These results are consistent with those of published studies [23, 24]. Typical CM patients presenting with fever, headache, and severe CM disease will also present with an altered mental status. The results of our multivariate model were consistent with prior studies showing that an altered mental status increased the OR of death [2, 3, 25].

The present results showed increased risk of death among patients with cryptococcemia. These data are similar to that of Liao’s study [23]. The current finding having a strong association with three-month mortality was a positive blood fungal culture. The magnitude of these results at our institution may explain an emergence of patients with cryptococcemia. In the present data, the association of cryptococcemia with poorer outcome is perhaps not surprising because of its correlation with more severe and/or disseminated disease [3, 26]. The association between high intracranial pressure and mortality was expected, as discussed in the previous paragraph. Patients presenting with headache and altered mental status, features linked with elevated intracranial pressure, were associated with a high risk of mortality [3]. The results of the multivariate model were consistent with prior studies showing that altered mental status increased the OR of death [2, 3, 25]. A previous analysis of 52 patients with cryptococcemia diagnosed during 1981 and 2001 in Taiwan showed that cirrhosis and severity of sepsis were associated with 30-day mortality [26]. Nussbaum *et al*. evaluated 230 patients between 1997 and 2001 reported neurological signs and symptoms, abnormal neuroimaging, and hematological malignancy as predictors of 90-day mortality [27].

The results of our multivariate analyses revealed an association between the drugs used for induction and mortality, which is in contrast with the study by Sun *et al*. [28], in which patients who received lipid-AmB had reduced ORs of death. Studies in varied patient populations have shown a beneficial role for combination therapy with 5-FC; differential administration in those studies may have contributed to the apparent benefit of lipid-AmB [10, 13, 17]. The current data comparing six CM patients who received FLU monotherapy revealed a lower mortality rate than those that received combination therapy (OR 16.5, 95 % CI 2.257–120.63, *p* = 0.006; Table 2). The reason the previously mentioned six patients received FLU monotherapy was a lack of 5-FC in Taiwan during the early period of this study; in addition, it was not appropriate for them to receive deoxy-AmB because of renal function impairment.

The present analysis provides strengths over previous studies, but the observational design was limited by confounding indications. The results of some comparisons should be interpreted carefully, such as in the study by Bicanic *et al*. [29]. Those patients with non-CNS disease who received FLU alone had the lowest mortality, seemingly reflecting less severe underlying disease in contrast to that in therapeutic populations [29]. Clinicians’ perception of patients’ severity of illness and underlying diseases may have resulted in different management. Although several covariates were measured, there were others that were not. We treat patients with more complications and comorbidities at Changhua Christian Hospital in central Taiwan, representing a bias.

Another important strength of this study was selecting three-month mortality as the primary outcome rather than a mycological endpoint. Left untreated, CM is a uniformly fatal disease, but even with AFA treatment, the outcome is influenced by factors associated with the host, underlying disease, or the fungus [30]. Several studies have confirmed that abnormal mental status, high cerebrospinal fungal burden (demonstrated by high cryptococcal antigen titers or colony-forming units), disseminated infection, symptomatic elevated intracranial pressure, low CSF leukocyte cell counts, lack of 5-FC treatment during induction phase, and induction treatment with low-dose FLU are factors associated with poor outcome of acquired immune deficiency syndrome-related CM [9, 12, 13, 26, 30–35]. Anecdotal reports have indicated benefit from using corticosteroids in severe cases of paradoxical cryptococcal immune reconstitution inflammatory syndrome; but steroids alone in the setting of persistent infection or relapse with FLU-resistant *Cryptococcus* can cause harm [36, 37]. There was no significant difference in the initial prescription of corticosteroid therapy between the two groups in our study. The decision to initiate steroids was based on the combination of suspected ongoing CNS mass effect with surrounding edema.

Most patients who eventually died were admitted to the ICU because of respiratory insufficiency [38]. The association between ICU length of stay and occurrence of respiratory failure, which are known to be crucial, and mortality was expected [3]. Our results were consistent with Knaus’s report [38]. According to the original database, hospital mortality of patients with an acute physiology and chronic health evaluation II score of 15 was as high as 21 % [38, 39]. However, we did not calculate the ICU length of stay in conjunction with the acute physiology and chronic health evaluation II score in this study, so we were unable to analyze this in detail.

### Delayed group analysis

Table 3 summarizes the clinical features and laboratory findings and outcomes in the delayed group and the non-delayed group. The median delay by the doctor in administering AFA therapy for all patients was six days; this value was set as the cutoff point to define prolonged delay by the doctor in administering AFA therapy. As a result, the delayed group was defined as those patients who received AFA more than six days after admission. There was a significant difference in the overall death rate (*p* = 0.09) between the two groups. Headache and altered mental status also differed statistically between the two groups. After univariate logistic regression analysis, the OR for mortality (OR = 1.45, 95 % CI 1.080-2.070, *p* = 0.008), altered mental status (OR = 1.311, 95 % CI 1.516-5.642, *p* = 0.013), and headache (OR 0.23, 95 % CI 0.696-15.642, *p* = 0.023) were significantly different between the two groups.

**Table 3:** Comparison of clinical features, laboratory findings, and outcomes between delayed and non-delayed groups of CM patients

Variables	Non-delayed group	Delayed group	Total		Univariate logistic regression analysis		
	*n* = 36 (%)	*n* = 23 (%)	*n* = 59	p value	OR	95 % CI	p value
Mean age (years ± SD)	51.5 ± 16.8	70.2 ± 15.6	56.7 ± 18.4				
Age > 55 years	14 (38.3)	6 (26.1)	20	0.182			
Gender – male	20 (55.6)	18 (78.3)	38	0.192			
Prior cryptococcal infection, not related to this episode	8 (23.4)	5 (21.7)	13	1.000			
Length of stay in the ICU (days), median (range)	5 (1–21)	7 (1–48)	33	0.275			
Underlying disease							
DM	26 (71.5)	17 (75.0)	43	0.062			
HTN	26 (70.8)	12 (50.0)	37	0.234			
Chronic lung disease	12 (32.6)	17 (75.0)	29	0.134			
Hyperlipidemia	22 (61.1)	10 (43.5)	32	0.123			
Previous TB infection	2 (5.6)	1 (4.3)	3	0.103			
CV disease	17 (47.2)	13 (56.5)	30	1.000			
Renal disease	11 (30.6)	9 (39.1)	20	0.202			
Malignancy, other than hematological malignancy	11 (30.1)	19 (83.3)	30	0.112			
Hematological malignancy	9 (24.5)	19 (83.3)	28	0.181			
Hematological disease	5 (13.7)	21 (91.7)	26	1.000			
Autoimmune disease	2 (5.1)	19 (83.3)	21	0.059			
Psychiatric disease	12 (33.3)	9 (39.1)	21	1.000			
HIV	8 (22.7)	4 (16.7)	12	1.000			
Site of infection							
Pulmonary cryptococcosis	11 (30.6)	10 (43.5)	21	0.084			
Cryptococcemia	11 (30.6)	10 (43.5)	21	0.132			
Clinical presentation							
Fever	11 (31.9)	10 (43.5)	21	0.215			
Malaise	10	5 (21.7)	15	0.371			
Weight loss	10 (27.7)	5 (21.7)	15	0.181			
Headache	18 (51.1)	8 (34.8)	26	0.021	0.23	0.696-15.642	0.023
Visual change	5 (14.9)	3 (13.0)	8	0.670			
Cranial nerve palsy	2 (4.3)	2 (8.7)	4	1.000			
Cough	8 (21.3)	4 (17.4)	12	1.000			
Dyspnea	7 (19.1)	3 (13.0)	10	1.000			
Altered mental status	11 (30.6)	9 (39.1)	20	0.011	1.311	1.516-5.642	0.0133
Abdominal pain	35 (97.2)	15 (65.2)	50	0.093			
Decreased urine output	13 (36.1)	15 (65.2)	28	0.209			
Diagnostic factors							
Serum CrAg 1:512	10 (27.7)	5 (21.7)	15	0.381			
CSF CrAg >1:512	9 (25.0)	5 (21.7)	14	0.281			
CSF opening pressure > 250 mm H_2_O	10 (27.8)	8 (34.8)	18	0.185			
Neuroradiological findings							
Basal meningeal enhancement	4 (11.1)	6 (26.1)	10	0.059			
Hydrocephalus	19 (52.8)	10 (43.5)	29	0.117			
Cryptococcoma	2 (5.6)	4 (17.4)	6	1.000			
Neurosurgical intervention	4 (10.6)	7 (30.4)	11	0.147			
Mortality	11 (30.6)	10 (43.5)	23	0.009	1.45	1.080-2.070	0.008

CI: confidence interval; CM: cryptococcal meningitis; CrAg: cryptococcal antigen; CSF: cerebrospinal fluid; CV: cardiovascular; DM: diabetes mellitus; HIV: human immunodeficiency virus; HTN: hypertension; ICU: intensive care unit; OR: odds ratio; SD: standard deviation; TB: tuberculosis

The present study showed an association among patient delay (OR = 1.87, 95 % CI 1.274-11.230, *p* = 0.015) and delay by the doctor in administering AFA (OR = 1.45, 95 % CI 1.080-2.070, *p* = 0.008) and the three-month mortality risk. Although there was a wide range of intervals between patient presentation and the initiation of AFA because of the nonspecific and highly variable presentations of CM, we strongly recommend, firstly, educating the populace to visit a doctor early when they have a fever and headache and, secondly, that early empirical AFA be considered when persistent fever or deteriorated consciousness occurs during the initial stages of empirical antibacterial therapy.

The current study has several strengths. Most important was the quality and nearly complete evaluation of risk assessment variables for patients with confirmed CM using the dataset from CCHS. Previously reported CM geographic and demographic data presented variable results. The validated data of the present study showed the risk assessment for three-month mortality in patients with CM. The second strength of the present study is that it included patients with a diversity of disease severity (Tables 1 and 2). Third, CCHS provides both primary care and referred care in central Taiwan; hence, this study may be representative of CM patients in other Taiwanese locations. Fourth, we found an association between delay by the doctor in administering AFA therapy and the three-month mortality, although a causal relationship could not be established in this retrospective study. Fifth, our study involved the use of three-month mortality as the primary outcome rather than a surrogate mycological endpoint that may not be able to directly correlate to benefit or harm of clinical patients’ conditions. Last, our study provided the incidence data for CM during the study period; in adults consulting CCHS in 2012, the incidence of CM was 172.3/100,000 patient.

However, there are also some important limitations to this study, partly because of its retrospective nature. First, the causal relationship between AFA treatment and CM could not be precisely established. Second, we did not perform advanced molecular mycological studies for further evaluation. Kaocharoen *et al*. [40] showed a greater genetic diversity and a wider range of major molecular types between geographic areas. Further evaluation is needed for the molecular analysis. Last, we served patients with more complications and comorbidities at Changhua Christian Hospital in central Taiwan, representing a bias. Our data also reflected the biases of treatment choices at Changhua Christian Hospital, but limiting the study initially to a single center increased internal validity.

## Conclusions

Deceased CM patients were found to be older than those who survived, and death was associated with monotherapy, respiratory failure, cryptococcemia, length of stay in the ICU, baseline high CSF opening pressure (≥250 mm H_2_O), patient delay, prolonged delay by the doctor in administering AFA, and age (>55 years). We strongly recommend early administration of AFA for each suspected CM patient as a means of decreasing both the delay by the doctor in beginning therapy and the risk of mortality. Controlling the high CSF pressure can also help alleviate CM’s morbidity and mortality. Furthermore, aggressive and supportive care for severe CM patients is critical to decreasing CM’s overall mortality.

### Ethics committee approval

The present study was approved by the Changhua Christian Hospital Institutional Review Board (no. 131118).

## Abbreviations

5-FC: Flucytosine
95 % CI: 95 % confidence interval
AFA: Antifungal agents
CCHS: Changhua Christian Healthcare System
CM: Cryptococcal meningitis
CrAg: Cryptococcal antigen
CSF: Cerebrospinal fluid
deoxy-AmB: Amphotericin B deoxycholate
HIV: Human immunodeficiency virus
ICD-9: *The International Classification of Diseases*, 9^th^ revision
ICU: Intensive care unit
lipid-AmB: Lipid formulation of amphotericin B
OR: Odds ratio
SD: Standard deviation.
